# Level of inequality and the role of governance indicators in the coverage of reproductive maternal and child healthcare services: Findings from India

**DOI:** 10.1371/journal.pone.0258244

**Published:** 2021-11-12

**Authors:** Sumirtha Gandhi, Tulasi Malini Maharatha, Umakant Dash, Suresh Babu M.

**Affiliations:** 1 Bengaluru Dr. B.R. Ambedkar School of Economics, Karnataka, India; 2 Department of Humanities and Social Sciences, Indian Institute of Technology, Chennai, India; Institute for Human Development, INDIA

## Abstract

**Background:**

Diligent monitoring of inequalities in the coverage of essential reproductive, maternal, new-born and child health related (RMNCH) services becomes imperative to smoothen the journey towards Sustainable Development Goals (SDGs). In this study, we aim to measure the magnitude of inequalities in the coverage of RMNCH services. We also made an attempt to divulge the relationship between the various themes of governance and RMNCH indices.

**Methods:**

We used National Family Health Survey dataset (2015–16) and Public Affairs Index (PAI), 2016 for the analysis. Two summative indices, namely Composite Coverage Index (CCI) and Co-Coverage (Co-Cov) indicator were constructed to measure the RMNCH coverage. Slope Index of Inequality (SII) and Relative Index of Inequality (RII) were employed to measure inequality in the distribution of coverage of RMNCH. In addition, we have used Spearman’s rank correlation matrix to glean the association between governance indicator and coverage indices.

**Results & conclusions:**

Our study indicates an erratic distribution in the coverage of CCI and Co-Cov across wealth quintiles and state groups. We found that the distribution of RII values for Punjab, Tamil Nadu, and West Bengal hovered around 1. Whereas, RII values for Haryana was 2.01 indicating maximum inequality across wealth quintiles. Furthermore, the essential interventions like adequate antenatal care services (ANC4) and skilled birth attendants (SBA) were the most inequitable interventions, while tetanus toxoid and Bacilli Calmette- Guerin (BCG) were least inequitable. The Spearman’s rank correlation matrix demonstrated a strong and positive correlation between governance indicators and coverage indices.

## 1. Background

Measuring the coverage of Reproductive, Maternal, New-born and Child Health (RMNCH) services is prerequisite to monitor the progress towards Sustainable Development Goals (SDGs) which superseded the Millennium Development Goals (MDGs). These goals embody the attainment of universal coverage of essential and preventive interventions as its primal focus. Universal health coverage (UHC) necessitates achievement of equity along with the overall increase in the coverage of RMNCH interventions and it is purported that coverage measures play a crucial role in suggesting policy initiatives striving to achieve universal coverage and SDGs [1].

There is a copious amount of evidence depicting a considerable progress in the performance of maternal and child health indicators. For instance, the number of global maternal deaths reduced from 532000 to 295000 between 1990 and 2017 and under-five deaths plunged from 12.7 million (1990) to 5.2 million (2019) [2]. Despite such greater strides, only 9 countries in the world achieved Millennium Development targets pertaining to maternal and child health [3]. Moreover, 15 percent of the world’s maternal deaths are contributed by India alone [3]. According to the estimates of Sample Registration System (SRS), the Maternal Mortality Ratio (MMR) of India plummeted from 556 (1990) to 113 (2016–18) [4, 5]. The under-five mortality ratio fell from 115 (1990) to 36 (2018) [6, 7]. But the progress did not penetrate evenly across different segments of population. This is reflected in burgeoning disparity across different geographical contours. For instance, the MMR in Empowered Action Groups (EAG) states were 520 in 1997–98, 375 in 2004–05 and 161 in 2016–18 [4, 8]. Whereas in the southern states, the corresponding estimates were 187 (1997–98), 149 (2004–05), and 67 (2016–18) [4, 8].

These emerging inequities calls for a diligent measurement of RMNCH at national as well as sub-national levels. This task has been extensively conducted across different countries [9–11]; and the previous studies have used Composite Coverage Index (CCI) or standalone indicators of RMNCH to assess the coverage [12, 13]. Implementation of any health program/scheme/intervention requires a supportive governance system to reach the targeted population successfully. Some of the existing studies have measured the relationship between governance indicators and inequalities in RMNCH in low and middle-income countries [14–16]. However, studies measuring the coverage of RMNCH interventions are scarce in India [17, 18] and none to our knowledge have measured the association between governance and RMNCH indicators.

CCI is the weighted average of eight different indicators ranging across the entire lifecycle of pregnancy and childhood care, it is insensitive to sampling variability and has a strong association with maternal and child health outcomes. Co-Coverage indicator (Co-Cov), an alternative indicator has been used to estimate the extent of coverage- it provides an assessment of range of preventive public health intervention and also enumerates the percentage of woman/children receiving crucial preventive interventions [19].

Researcher have however argued that, estimation of CCI along with the Co-Cov is pertinent to comprehend the status of RMNCH in wholesome [20]. Measurement of CCI and Co-Cov across different wealth quintiles and geographical regions provide crucial information about which groups of women and children are lagging behind in terms of RMNCH coverage. One of the contributions of this paper is the usage of two summary indices namely, CCI and Co-Cov to measure inequality in RMNCH coverage. Additionally, we have gauged the association between different dimensions of Overall Governance Index (OI) and CCI/Co-Cov of RMNCH services. This exercise will enable us to understand which indicator of OI is influencing RMNCH services more and which indicator has a lesser influence and hence enables us to understand which Governance indicator has to be concerted more attention to observe greater improvement in the coverage of RMNCH interventions.

## 2. Methods

### 2.1 Data

For the empirical analysis we used National Family Health Survey (NFHS) conducted in 2015–16 and Public Affairs Index (PAI), 2016. NFHS employs a two-stage stratified random sampling method and interviewed women belonging to the reproductive age group (15–49 years). Total sample of this survey is 601,509 households, 699,686 eligible women, and 259,627 children (younger than 5 years). Data on Public Affairs Index (PAI) discerning the quality of governance, is extracted from the Public Affairs Centre (PAC) [21]. This centre adopted a multifaceted approach considering 10 distinct theme to construct a governance indicator. These themes were normally categorised into rule based and performance based measures (Further details are provided in S1 Data). To ensure the compatibility of PAI with NFHS (2015–16), we used PAI indices published in 2016 for all states, except Telangana and Andhra Pradesh because the information regarding these two states were collated into one (collected before territorial bifurcation). Hence, for these two states we resorted to PAI (2017).

### 2.2 Methodology

We defined a comprehensive list of RMNCH indicators and constructed two summative indices namely, CCI and Co-Cov. These indices provide complete information about RMNCH interventions by avoiding information overload.

CCI is constructed using four distinct themes swaying across different stages of the continuum of maternal and child healthcare services, starting from ***reproductive health*** (usage of modern contraceptives (FPS)), ***maternal health care services*** (four or more antenatal visits (ANC4), Skilled Birth Attendants (SBA)), ***new-born and child healthcare vaccination*** (3 doses of Diphtheria, Pertussis and tetanus vaccine (DPT3), measles vaccination (MSL), Bacilli Calmette- Guerin (BCG) vaccination (BCG)) and ***utilisation of healthcare services among children’s*** diagnosed with pneumonia (CPNM) and Oral rehydration therapy and other fluids for children with diarrhoea (ORT). A detailed description of these variables is provided in the [S2 Data] supplementary document.

Co-Cov indicator entails a range of preventive public health interventions—ANC4, Tetanus Toxoid dosage during pregnancy, SBA, BCG vaccination, DPT3 vaccination, Measles vaccination and household’s access to improved drinking water [19, 20]. The Co-Cov is defined as the number of interventions utilised by each woman and child pair. It is a binary indicator coded as 1 for (adequate utilisation) woman and child pair receiving 6 or more interventions of the aforementioned interventions and 0 for those availing less than 6 interventions (inadequate utilisation).

These two indices have been broadly used in the literature to examine the overall performance of different geographical regions such as, country/state/specific region in the provisioning of maternal and child health care services [9, 18, 22]. In the present study, we have adopted the following formula for the construction of CCI [Input file indicating the definition and computation of CCI is provided in S3 Data].


$$CCI=\frac{1}{4}\left(FPS+\frac{SBA+ANC}{2}+\frac{2DPT3+MSL+BCG}{4}+\frac{\left(ORT+CPNM\right)}{2}\right)$$

The above equation demonstrates a weighted average mean of eight essential interventions by assigning equal weightage for each of its component [16, 18, 22].

This paper aims to undertake an inequality analysis of RMNCH coverage across distinct wealth horizons. Wealth Index have been widely used to measure economic status of the individuals [23]. It is defined in the form of quintiles, where 1^st^ quintile (Q1) represents the poorest 20 percent of the population and last quintile (Q5) reflects the richest 20 percent of the population. Previous studies have predominantly used absolute and relative inequality methods to demonstrate inequality in the coverage of RMNCH. The absolute inequality (Q5-Q1) ascertained the magnitude of differences in the coverage of maternal and child health services between the richest and poorest quintile. Whereas, relative inequality (Q5/Q1) explains the ratio/relative differences in the coverage of maternal and child health services between the richest and poorest quintile.

Although these two methods hold an advantage of easy interpretability from a layman’s perspective, they suffer from certain serious drawbacks. First, when the stratification within a concerned sub-group changes, the level of inequality also varies. Second, the variations in the overall coverage levels do not necessarily represent a similar variation across wealth quintiles. In other words, the highest and lowest coverage among top and bottom quintile population do not necessarily represent highest and lowest coverage in the overall population. Finally, these two techniques do not take into account the intermediate wealth quintiles such as 2^nd^ quintile (Q2), 3^rd^ quintile (Q3) and 4^th^ quintile (Q4). We have adopted two advanced measurement techniques such as Slope Index of Inequality (SII) and Relative Index of Inequality (RII) to overcome the shortcomings encountered in previous techniques [9, 24].

To compute inequality for CCI and Co-Cov, we gathered indicator-wise information for each quintile by incorporating their respective weights. For each quintile, we followed a systematic approach encompassing five stages of computation. In the *first stage*, we used the individual level dataset and grouped it across quintiles for each of the indicator, in the *second stage*, quintile wise proportions, were computed for each indicators. These proportions were cumulatively added in the *third stage* and in the *fourth stage*, we divided these cumulative proportions by 2 to compute mid-point values. In the *final stage*, the logistic regression was conducted between the mid-point of the population in each quintile and indicators (CCI/Co-Cov) to estimate SII and RII.

The relationship between OI and RMNCH coverage inequality indices (CCI & Co- Cov) were captured by constructing Spearman’s rank correlation matrices. Detailed insights were gathered by conducting correlation analysis across each theme of governance indicator and coverage indicators. The governance indicator and its indices values are calculated by PAC and details of each of the index values across states are provided in appendix section [S4 Data].

We adhered to the Strengthening the Reporting of Observational Studies in Epidemiology (STROBE) guidelines published by the PLOS medicine and structured our manuscript accordingly [25]. We employed secondary dataset and hence ethical consideration was not required. Internal consistency of the indicators used for the construction of indices were ensured through Cronbach’s alpha test. All the statistical analyses were performed using Excel, Stata version 15.0, R version 3.5.2 and GeoDa 1.14.0 (shape file can be availed from Survey of India).

## 3. Results

Table 1 presents the descriptive statistics of the sample pregnant women for major Indian states across wealth quintiles. In Bihar (80.93) and Jharkhand (72.43) maximum percentage of sampled pregnant women hailed from poor/ poorest quintile. In West Bengal, Madhya Pradesh and Uttar Pradesh, nearly 55 to 60 percent belonged to bottom quintile. In Tamil Nadu, Karnataka, Andhra Pradesh and Telangana, around 70 to 80 percent of the population belonged to non-poor categories. Nearly 95 percent of the sampled population of Kerala and Punjab belonged to middle income quintile or richer quintile population.

**Table 1:** Descriptive statistics of sampled pregnant women across major states of India: Quintile-wise disaggregation.

States	Poorest		Poor		Middle		Rich		Richest	
	[N]	[%]	[N]	[%]	[N]	[%]	[N]	[%]	[N]	[%]
Andhra Pradesh	295	(04.66)	1047	(16.54)	2252	(35.57)	1820	(28.75)	917	(14.48)
Bihar	13489	(57.57)	5473	(23.36)	2477	(10.57)	1531	(06.53)	459	(01.96)
Gujarat	875	(10.90)	1558	(19.41)	1928	(24.02)	1797	(22.39)	1867	(23.26)
Haryana	113	(02.58)	379	(08.64)	870	(19.84)	1254	(28.59)	1770	(40.36)
Himachal Pradesh	15	(01.79)	86	(10.24)	187	(22.26)	316	(37.62)	236	(28.10)
Karnataka	498	(06.36)	1556	(19.88)	2237	(28.58)	2153	(27.51)	1383	(17.67)
Kerala	11	(00.32)	55	(01.58)	405	(11.67)	1410	(40.62)	1590	(45.81)
Madhya Pradesh	4484	(35.35)	2942	(23.19)	1985	(15.65)	1714	(13.51)	1559	(12.29)
Maharashtra	1538	(09.68)	2960	(18.62)	3812	(23.99)	4354	(27.40)	3229	(20.32)
Odisha	2530	(40.10)	1622	(25.71)	1168	(18.51)	687	(10.89)	303	(04.80)
Punjab	25	(00.76)	138	(04.22)	451	(13.79)	767	(23.45)	1890	(57.78)
Rajasthan	2309	(21.35)	2679	(24.77)	2256	(20.86)	1873	(17.32)	1697	(15.69)
Tamil Nadu	311	(02.77)	1647	(14.65)	3344	(29.75)	3695	(32.87)	2244	(19.96)
Uttar Pradesh	10688	(33.68)	7650	(24.11)	5434	(17.12)	4290	(13.52)	3671	(11.57)
West Bengal	3402	(26.48)	4174	(32.49)	2543	(19.80)	1879	(14.63)	848	(06.60)
Telangana	327	(06.06)	862	(15.97)	1318	(24.41)	1649	(30.54)	1243	(23.02)

**Fig 1:**
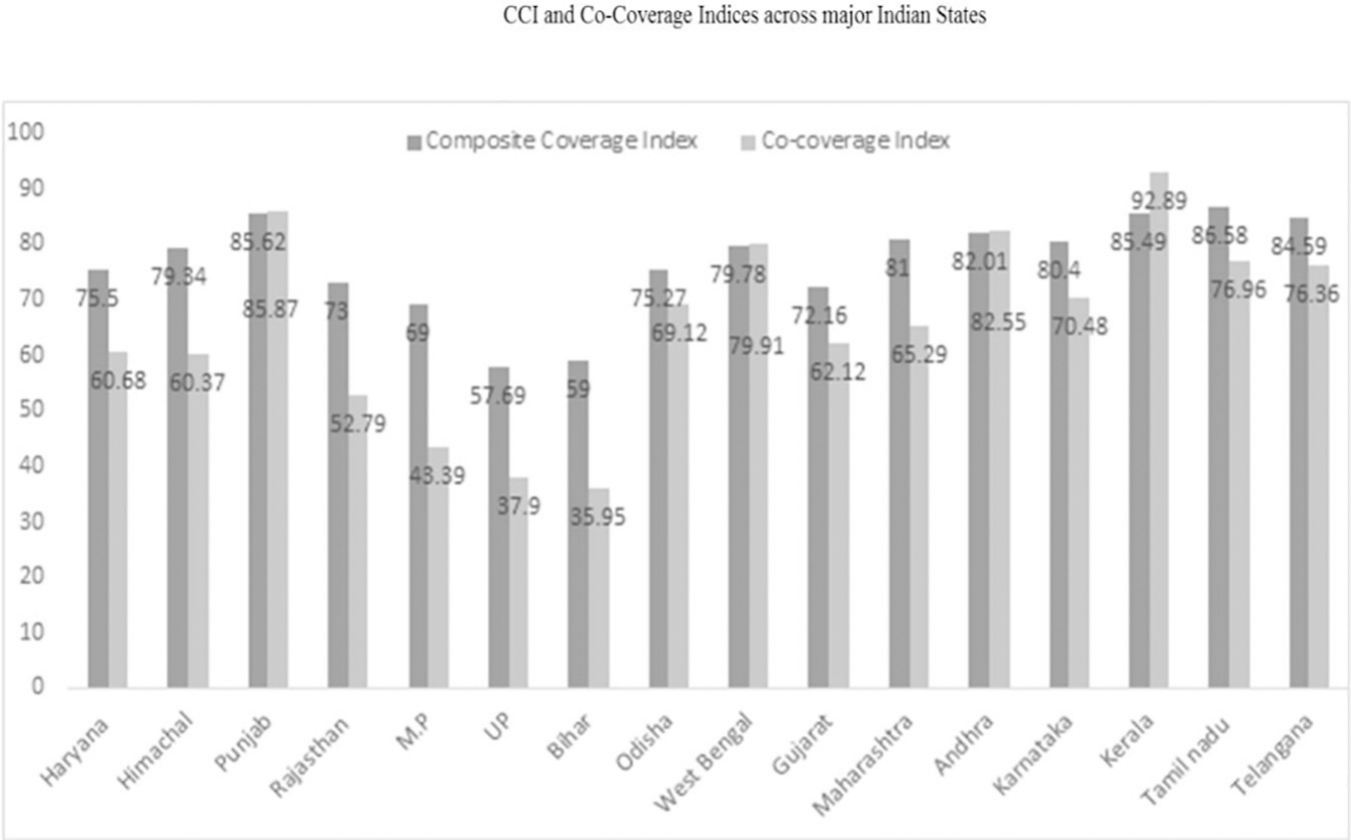


Fig 1 demonstrates the distribution of CCI and Co-Cov indices across major Indian states. The coverage of CCI is higher compared to Co-Cov indicator for all the states except Kerala, Punjab and Andhra Pradesh. The coverage of Co-Cov indicates disparate inequality across the states of India. According to this indicator, Kerala topped in terms of its performance. While Punjab, Uttar Pradesh (37.9 percent) and Bihar (35.95 percent) remained the back runners. The coverage of CCI is highest in Tamil Nadu (86.58 percent) and Punjab (85.62 percent), whereas in Bihar (59 percent) and Uttar Pradesh (57.69 percent), the performance of these indicators were abominably poor.

Table 2 indicates the absolute (Q5-Q1) and relative (Q5/Q1) inequality in the coverage of CCI and Co-Cov. The positive (negative) value of absolute difference discerns higher coverage of RMNCH interventions in richest (poorest) quintile. While, the ratio difference value greater than unity reflects that the utilisation of RMNCH services by richest quintile population is comparatively higher than the poorest quintile. In Haryana, the absolute difference between richest and poorest quintile was 37.2 percent, while in Karnataka absolute variation was comparatively negligible. The levels of absolute inequality was more than 20 percent in the states of Haryana, Uttar Pradesh, Gujarat, Kerala, Andhra Pradesh and Telangana. On the other hand, in West Bengal, Tamil Nadu and Maharashtra percentage of absolute inequalities were around less than 10 percent.

**Table 2:** Absolute and relative differences in the coverage CCI and Co-Cov indicators across major Indian states.

States	CCI		Co-Coverage	
	[(Q5-Q1)]	[(Q5/Q1)]	[(Q5-Q1)]	[(Q5/Q1)]
Himachal Pradesh	13.4	1.189	40.51	2.19
Punjab	8.20	1.103	26.65	1.41
Haryana	37.2	1.846	54.51	3.96
Rajasthan	19.5	1.309	44.34	2.30
Uttar Pradesh	24.5	1.504	42.57	2.70
Bihar	16.7	1.301	41.62	2.45
West Bengal	08.4	1.107	22.84	1.33
Odisha	10.6	1.149	15.61	1.26
Madhya Pradesh	16.9	1.265	46.65	2.74
Gujarat	22.3	1.380	53.69	2.89
Maharashtra	9.50	1.128	23.79	1.49
Andhra Pradesh	21.9	1.329	24.06	1.36
Karnataka	-04.6	0.945	-1.08	0.98
Kerala	20.5	1.306	10.07	1.12
Tamil Nadu	8.70	1.110	13.13	1.20
Telangana	21.0	1.300	32.03	1.59

The relative differences provide additional insights by divulging the degree of unfairness between richest and poorest quintile. The value of relative differences were greater than unity for all Indian states with an exception being Karnataka (0.94). Table 2 demonstrates highest pro-rich inequality in Haryana (18.4) indicating greater utilisation amongst richest quintile compared to the poorest quintile.

The distribution of Co-Cov indicators discerned an existence of disproportionate spread of absolute inequalities across wealth quintiles in Haryana, Gujarat and Madhya Pradesh. The states of Kerala, Tamil Nadu and Odisha experienced an absolute inequalities of less than 20 percent. Besides, Karnataka stands out as an anomaly with a negative value of 1.08, representing slightly higher coverage amongst the lowest quintile compared to highest quintile. Relative inequality ratio was greater than 2 in Haryana, Himachal Pradesh, Rajasthan, Uttar Pradesh, Madhya Pradesh and Gujarat. In Tamil Nadu and Kerala, relative inequalities were 1.20 and 1.12 respectively. Karnataka, boasted a relative score of 0.98 informing that the coverage is higher for the poorest quintile compared to their wealthier counterparts. To collect additional insights, we have computed the values of SII and RII which encompasses the cumulative distribution by considering all wealth quintiles.

Table 3 provides the distribution of levels of inequalities across different socio-economic categories. Overall, the level of inequality was invariably high for central and northern regions except Punjab. For instance, the SII discerning the average difference in utilisation across wealth quintiles are outrageously high in Haryana and Uttar Pradesh. On the other hand, the states of Karnataka demonstrated lower levels of SII. The RII scores indicating the average ratio of utilisation across wealth quintiles was highest in Haryana with a score value of 2.01. On the other hand, in the states of Punjab, Tamil Nadu and West Bengal hovered around an RII score of 1.

**Table 3:** SII and RII of composite coverage index across major Indian states.

Region	States	SII	(95% CI)	RII	(95% CI)
*Southern Region*	Andhra Pradesh	0.20	(0.05–0.35)	1.29	(1.02–1.57)
	Karnataka	-0.06	(-0.08–0.05)	0.93	(0.90–0.95)
	Kerala	0.25	(0.16–0.33)	1.37	(1.21–1.54)
	Tamil Nadu	0.08	(0.00–0.16)	1.10	(0.99–1.21)
	Telangana	0.21	(0.11–0.31)	1.30	(1.13–1.47)
*Eastern Region*	Bihar	0.21	(0.20–0.22)	1.39	(1.37–1.42)
	Odisha	0.11	(0 .07–0.16)	1.16	(1.09–1.23)
	West Bengal	0.10	(0.06–0.14)	1.13	(1.07–1.19)
*Western Region*	Maharashtra	0.14	(0.09–0.18)	1.19	(1.13–1.25)
	Gujarat	0.26	(0.23–0.29)	1.46	(1.39–1.53)
*Northern Region*	Haryana	0.43	(0.34–0.52)	2.01	(1.64–2.38)
	Himachal	0.17	(0.12–0.22)	1.25	(1.17 - 1.33)
	Punjab	0.10	(0.08–0.12)	1.13	(1.10–1.16)
	Rajasthan	0.23	(0.19–0.26)	1.37	(1.31–1.43)
*Central Region*	Madhya Pradesh	0.20	(0.18–0.23)	1.33	(1.28–1.37)
	Uttar Pradesh	0.29	(0.25–0.33)	1.64	(1.54–1.75)

Table 4 illustrates the state-wise estimation results of SII and RII for Co-Cov in India. The states of Gujarat (56 percent), Madhya Pradesh (52.4 percent) and Rajasthan (48.5 percent) experienced greater levels of inequality. Whereas, in Karnataka and Kerala, the distribution of Co-Cov was close to equality. The RII was extremely high in Madhya Pradesh and Uttar Pradesh, while the states of Karnataka, Kerala and Tamil Nadu exhibited lower levels of inequality.

**Table 4:** SII and RII of Co-Coverage indicator across major Indian states.

Region	States	SII	(95% CI)	RII	(95%)
**Southern**	Andhra Pradesh	0.20	(0.14–0.26)	1.28	(1.18–1.38)
	Karnataka	-0.02	(-0.09–0.06)	0.98	(0.88–1.08)
	Kerala	0.01	(-0.03–0.06)	1.01	(0.96–1.07)
	Tamil Nadu	0.10	(0.05–0.14)	1.14	(1.07–1.20)
	Telangana	0.25	(0.17–0.33)	1.40	(1.24–1.56)
**Eastern**	Bihar	0.35	(0.32–0.37)	2.72	(2.49–2.94)
	Odisha	0.27	(0.23–0.31)	1.50	(1.40–1.59)
	West Bengal	0.29	(0.25–0.34)	1.47	(1.37–1.58)
**Western**	Maharashtra	0.24	(0.18–0.30)	1.45	(1.32–1.59)
	Gujarat	0.56	(0.52–0.60)	2.80	(2.52–3.09)
**Northern**	Haryana	0.40	(0.36–0.45)	2.03	(1.84–2.21)
	Himachal	0.44	(0.36–0.51)	2.18	(1.84–2.52)
	Punjab	0.26	(0.21–0.31)	1.38	(1.28–1.48)
	Rajasthan	0.49	(0.46–0.51)	2.73	(2.54–2.92)
**Central**	Madhya Pradesh	0.52	(0.50–0.55)	3.81	(3.53 - 4.09)
	Uttar Pradesh	0.43	(0.41–0.45)	3.35	(3.14–3.56)

The variation in SII and RII in the two indices across the states are mainly attributed to the changes in the two main factors. First, the utilisation of modern contraceptives and second, the utilisation of child health services. For instance, the inequality (SII and RII) for CCI is much higher in Kerala than the inequality for Co-Cov in the state. The differences is mainly due to the poor utilisation of child health care services in Kerala. Moreover, the disparity between the poorest and richest quintile population is quite prominent.

**Fig 2:**
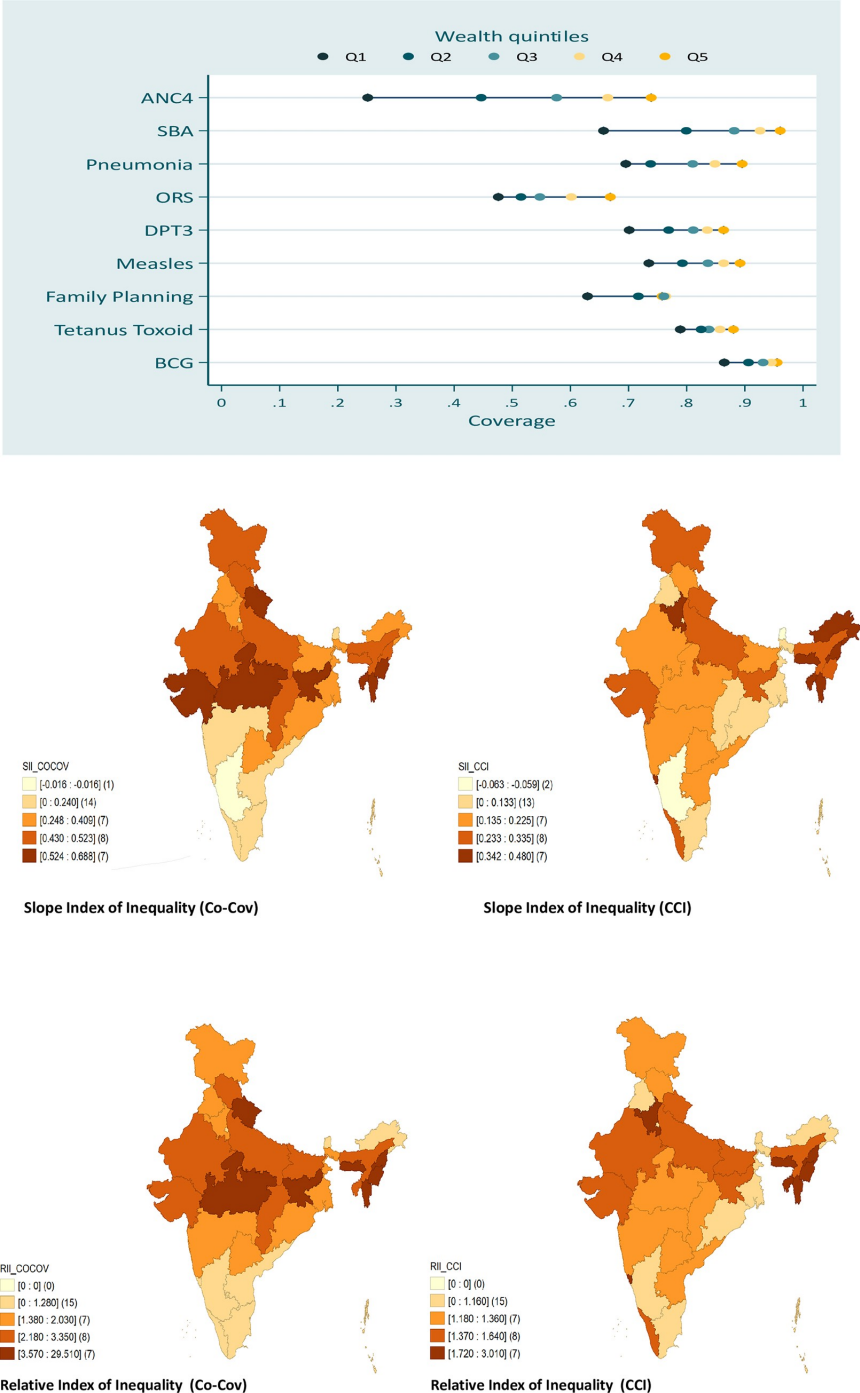


Fig 2A depicted the quintile wise variation in the coverage of RMNCH interventions through five-dot plot also known as equi-plot diagram. This figure indicates the coverage of individual RMNCH interventions across wealth quintiles. Y-axis represents the individual intervention of RMNCH and X-axis demonstrates proportion of the coverage of interventions disaggregated into quintiles. This method of diagrammatic representation disentangles the levels of inequalities in the coverage of RMNCH interventions into three distinct groups—linear, top and bottom level inequalities. If the estimated values of different quintiles are placed in equi-distance, then the distribution indicates linearity. Top and bottom inequalities explain the situation where the distribution is concentrated either in top-most quintile population or bottom quintile [14, 26].

Crucial maternal health interventions such as ANC4 and SBA were the most inequitable ones, while consumption of tetanus toxoid injections by pregnant women and BCG vaccination for children recorded lower levels of inequalities. It is important to notice that the coverage of essential interventions like ANC4, DPT3, Family Planning, Measles, and SBA manifested bottom level inequalities, in other words, the coverage of these interventions were lesser for lower quintile population compared to their richer counterparts. While, child-hood related behaviours such as seeking treatment for pneumonia and diarrhoea appeared to have linear and top level inequalities. The state level variations in terms of SII and RII for CCI and Co-Cov are mapped in Fig 2B.

### 3.1 Inter-state disparity across RMNCH interventions

Table 5 captured inter-state disparity across RMNCH interventions. Evidently, interventions necessitating frequent patient-provider consultations demonstrated maximum disparity. In India, ANC4 is the most inequitable intervention with an absolute gap of 49 percent (Q5-Q1). Similarly, DPT3, a child-related vaccination entailing three frequent visits to the facility demonstrated the largest absolute inequality of 16 percent. Despite garnering enormous attention from central and respective state governments, the coverage of SBA remains unevenly distributed with a maximum utilisation undertaken richest quintile population while poorest quintile population slogging much behind.

**Table 5:** Absolute gap in RMNCH coverage indicators across major states.

Region	Country/ States	ANC4	SBA	FP	BCG	DPT3	Measles
	India	0.49	0.30	0.09	0.13	0.16	0.16
***Southern Region***	Andhra Pradesh	0.21	0.17	-0.06	0.08	0.18	0.22
	Karnataka	0.02	0.04	-0.14	-0.04	-0.08	0.00
	Kerala	0.07	0.00	-0.01	-0.02	0.03	0.05
	Tamil Nadu	0.04	0.05	-0.05	0.03	0.41	0.13
	Telangana	0.22	0.24	-0.02	0.04	0.11	0.04
***Eastern Region***	Bihar	0.44	0.31	0.13	0.08	0.10	0.14
	West Bengal	0.24	0.30	-0.12	-0.04	-0.07	-0.02
	Odisha	0.20	0.17	-0.02	0.04	0.07	0.08
***Western Region***	Gujarat	0.46	0.28	-0.01	0.25	0.40	0.37
	Maharashtra	0.23	0.22	-0.05	0.11	0.19	0.12
***Northern Region***	Himachal Pradesh	0.44	0.41	-0.04	0.02	0.08	0.09
	Haryana	0.43	0.52	0.37	0.26	0.47	0.46
	Punjab	0.27	0.22	-0.05	0.05	0.04	0.02
	Rajasthan	0.39	0.21	0.09	0.17	0.25	0.26
***Central Region***	Uttar Pradesh	0.48	0.30	0.19	0.14	0.26	0.21
	Madhya Pradesh	0.45	0.31	-0.03	0.11	0.25	0.17

State-level mapping of ANC4 revealed widespread disparity—Uttar Pradesh indicated an absolute disparity of 48 percent, while Karnataka, recorded an absolute disparity of 1.6 percent. For DPT3, Haryana demonstrated a highest pro-rich distribution with an absolute inequality of 47 percent, while Karnataka (-0.08) exhibited a pro-poor distribution. Absolute inequality for SBA utilisation in India was 30 percent. Amongst the states, Haryana experienced maximum disparity with an absolute gap of 52 percent, while in Kerala the disparity was negligible.

### 3.2 Overall governance as a contextual factor for the coverage of RMNCH interventions

This section illustrates an empirical relationship between overall governance indicator (OI) and RMNCH interventions. Further, we enumerated the relationship between RMNCH interventions and different themes of OI to capture relationship associated with each theme [Details of each of the theme are provided in S2 Data].

**Fig 3:**
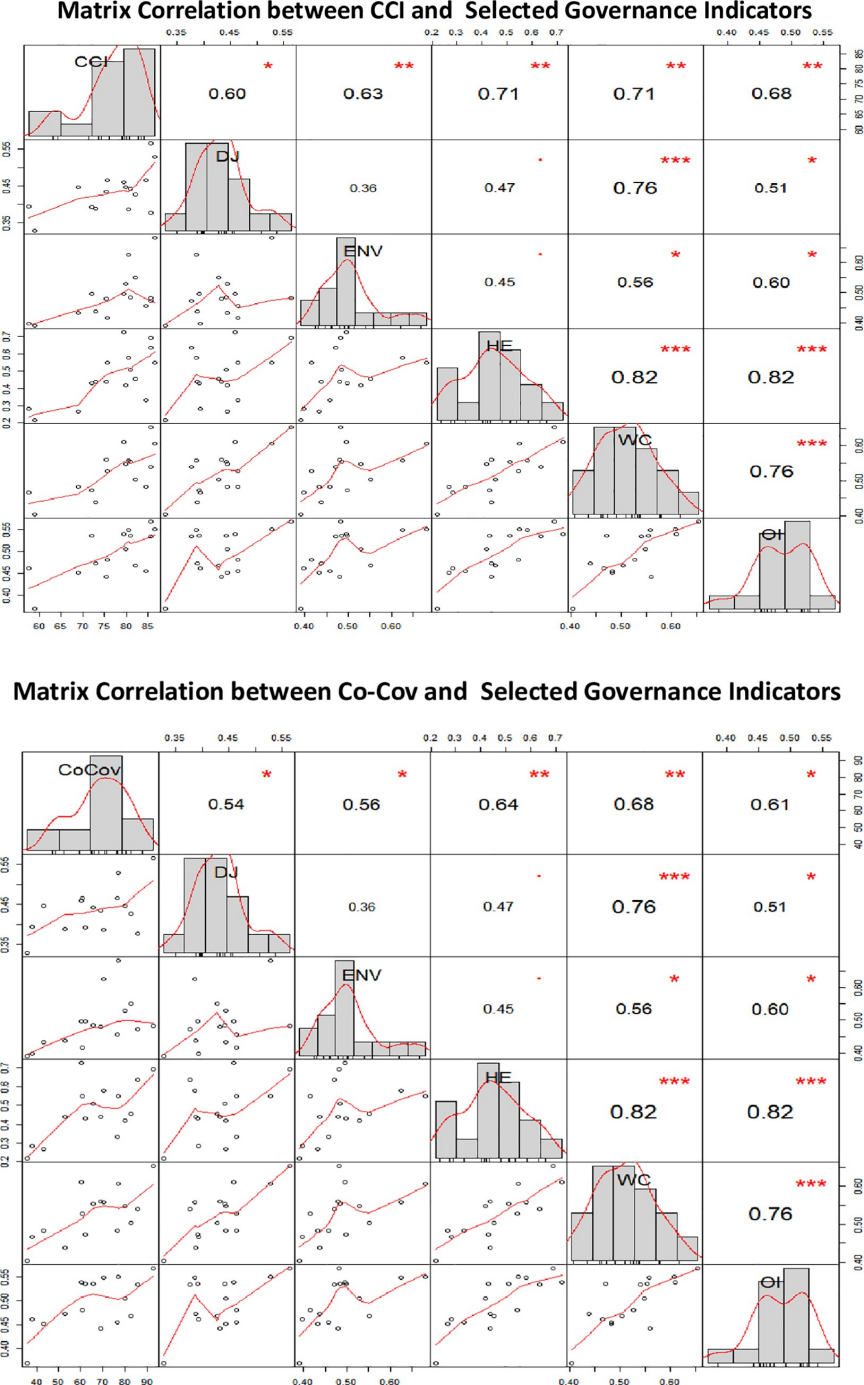


The results of Spearman’s correlation matrix indicate a strong and positive correlation between CCI and OI (Fig 3A) and between Co-Cov and OI (Fig 3B), with respective correlation coefficient values of 0.68 and 0.61. Although correlation analysis was conducted for each of the theme, the discussion is carried out only for themes indicating statistically significant association with CCI and Co-Cov (The correlation results for all the theme are provided in S5 Data). Theme-wise disaggregation demonstrated higher levels of positive correlation between Women and Child (WC) and Health and Education (HE) with RMNCH indicators. While Delivery of Justice (DJ) and Environmental factors (ENV) indicated a lower levels of positive correlation for CCI and Co-Cov.

**Fig 4:**
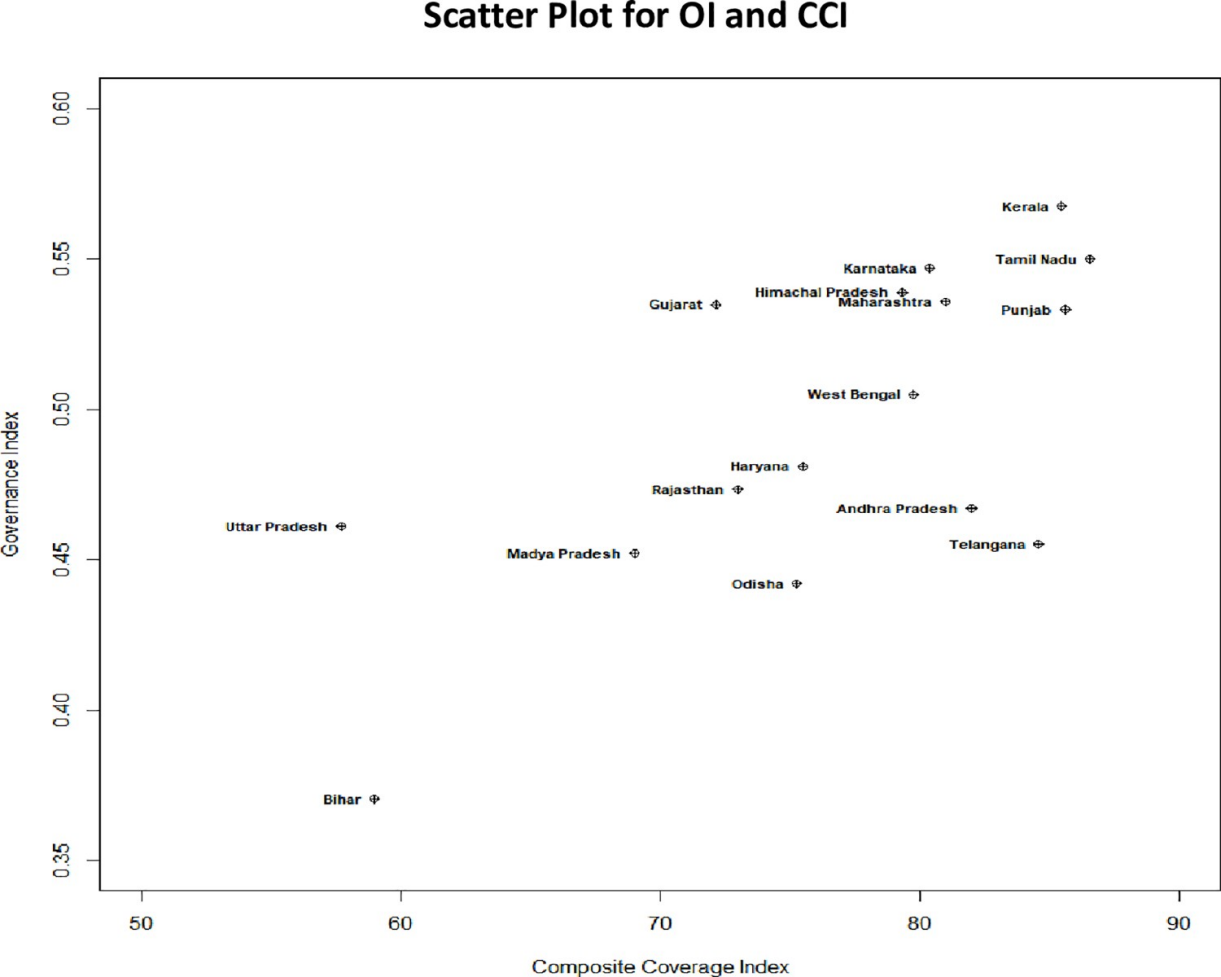


State-wise analysis revealed that the states performing better in terms of governance indicators, examples of which are Kerala, Tamil Nadu, Karnataka, Himachal Pradesh, Maharashtra and Punjab also performed better in terms of the coverage in CCI. While, the states of Bihar, Madhya Pradesh and Uttar Pradesh demonstrated poor performance in terms of both governance indicator and RMNCH coverage. Andhra Pradesh and Telangana turned out to be the exceptional ones, these states showed outstanding performance in terms of the coverage indicators despite having moderate governance scores. The details are depicted in Fig 4.

## 4. Discussion

The present study is an attempt to highlight new insights related to the coverage of CCI and Co-Cov and also discerns their correlation with OI.

### 4.1 Inequality in coverage of RMNCH services: Geographical regions

Findings of this study demonstrates an erratic disparities across and within the regions of India in terms of the coverage of RMNCH services. Similar conditions prevailed in other developing and developed countries [14, 18, 26]. In our study, the performance of RMNCH in southern states of India, were much higher than other states. The outstanding performance of southern region could be attributed to the prevalence of strong political commitment in the social sector, better awareness of health services within the community, greater levels of literacy among females, upgraded infrastructure of public health facilities and stronger delivery mechanisms [27–29]. It can hence be conferred that states concerting greater levels of attention in terms of the provisioning and distribution of health services have been successful in obtaining higher levels of RMNCH coverage [29].

Amongst the states experiencing pro-rich inequality, the absolute and relative disparity of CCI and Co-Cov are found to be lowest in Punjab. This could be mainly because, in Punjab, the primary health system had undergone a huge transformation, with most of the PHCs being upgraded to the Block PHC. The infrastructure and quality of these centres are comparable to the Community Health Centres (CHCs). Moreover, various new health facilities were created and managed by the local Governments [30, 31]. It can be concluded that relegation of authority to local governments might be an efficient strategy to reduce health inequality.

Surprisingly, in Karnataka, the utilisation of RMNCH services amongst the richest quintile population were proportionally lesser compared to poorest quintile. This could be explained by the proactive approach adopted by them to deliver quality healthcare services. The coverage of prenatal care services, delivery services, BEmOC and CEmOC facilities has increased across the districts indicating a concomitant reduction in the inequality of maternal and child healthcare services in Karnataka [27]. Moreover, a part of Karnataka which is also known as the “Silicon valley of India” accruing to its technological innovations might have transmuted its benefits to health sector in the form of promotion of digital health initiatives. The existence of digital monitoring mechanisms at the gram panchayat levels might have turned out to be an effective way of catering to the poorest quintile population. The role of technological innovations such as Digital Life Care, e-hospital projects linking patients and facilities, mobile health clinics ensure increasing accessibility of health care services to remote areas in Karnataka [32].

Inequality in the coverage of CCI and Co-Cov was highest in Haryana reflecting an outrageously high levels of inequalities across its districts [17, 31]. The existing disparities in the districts of Haryana could be explained by the variations in demand side factors like place of residence and socio-demographic factors and also by supply side components such as health policies, programme implementation, infrastructure and governance [31].

### 4.2 Inequalities in RMNCH interventions: Standalone indicators

The coverage of maternal health interventions varied distinctly across wealth quintiles, whereas, child health related interventions were more or less equitably distributed across bottom and top quintiles. Our analysis revealed that, inequality in the coverage of ANC4 (Q5-Q1 = 49) is much higher compared to other interventions. However, enormous inequality was also encountered in the coverage of SBA across different wealth quintiles (Q5-Q1 = 30). Under-utilisation of ANC4 and SBA was by the poorest quintile population was mainly contributed by financial and accessibility barriers [33, 34].

The distribution of ANC4 is quite disparate across states. Highest level of inequality in ANC4 was witnessed in Uttar Pradesh (Q5-Q1 = 48), while lowest inequality was experienced in Karnataka (Q5-Q1 = 2). The lowest level of inequalities in Karnataka could be attributed by the democratic decentralisation framework adopted by this state [29]. Similarly, the distribution of the utilisation of SBA were erratic across the states, with Kerala surpassing other states and Haryana displaying a greatest level of inequality with an absolute gap of 52 percent. Evidence suggests that Kerala has better accessibility and availability of health care services [35]. The National Health Profile (2017) has shown that the availability of government healthcare facilities in Kerala is 1280, while Haryana has only 159 health facilities in place [35].

Furthermore, amongst the child health related interventions—DPT3 and Measles has the higher level of inequalities (Q5-Q1 = 16). Whereas, the utilisation of BCG vaccination demonstrated an equitable distribution across wealth horizons. This could be partly associated with the accessibility hinderances prevalent amongst the poorest quintile population when the utilisation entails multiple visits. Typically, utilisation of adequate ANC4 and DPT3 entails multiple visits to the facilities [12], whereas the utilisation of BCG shots requires a one-time visit to the facility. Furthermore, the programmatic interventions and outreach programs related to BCG vaccination have percolated to grassroot levels due to the early and strict initiatives undertaken by the Government of India. Since 1948, BCG has gathered incessant attention as it was the only preventive vaccination available to control Tuberculosis in India [36]. DPT and Measles acquired attention much later with the implementation of the National Immunisation Programme called Expanded Programme of Immunisation in 1978 which aimed at expanding the coverage of both the diseases to 80 percent. These targets were however revised to 100 percent in the year 1985 with the initiation of the Universal Immunisation Programme. Despite these efforts, the coverage of DPT3 and Measles had remained sub-optimal and inequitable. This could be due to dearth of trained personnel managing the programme at the national and state levels [36, 37].

### 4.3 Correlation between governance indicators and RMNCH services

We discovered a significant correlation between OI and coverage indicators. We found that governance indicators such as, HE and WC demonstrated strong and positive association with coverage indicators, our findings are comparable to the findings of sub-Saharan countries [15]. Previous studies have postulated that the effectiveness of governance indicators have strong association with coverage amongst the poorest quintile compared to the richest quintile [16].

The higher correlation of WC and coverage indicators might be due to the influence of primal components of WC such as government’s utilisation of JSY fund and percentage of women working population ratio. Greater utilisation of JSY fund would have a direct/ intended impact on delivery and post-delivery care services and indirect/ unintended impact on child immunisation services. This argument have also been supported by various researchers in the previous studies [37, 38]. It indicates a greater level of government investment in the form of cash benefit programs to ensure higher utilisation of RMNCH services. The women working population ratio also had a positive correlation with the coverage of RMNCH services, this could be explained by a potential financial freedom of women resulting from an increase in female workforce. Interestingly, both these factors increase the purchasing power of the women and their financial freedom [39].

Our analyses also showed that HE is directly associated with the coverage of RMNCH services. It might be due to indicators of HE such as educational expenditure as a percentage of Gross State Domestic Product (GSDP) and health expenditure as a percentage of GSDP. Access to education in general and women in particular is an essential building-block for increasing RMNCH coverage. Previous research studies have also reported a direct association of the contribution of Gross Domestic Product (GDP) per capita and coverage [16]. However, our findings suggest that the coverage of RMNCH is influenced by specific contribution of GSDP on health and education.

## 5. Strengths & limitations of the study

The strengths of our analysis include a combined usage of two summative indices namely CCI and Co-Cov. These indicators together hinge on providing comprehensive information about the coverage of RMNCH services. Compared to a standalone indicator, the usage of CCI and Co-Cov results in a lesser random variability and generates robust results with greater precision. In addition, we have used the SII and RII to estimate the level of inequality across wealth quintiles. These methods provide additional information on inequality by considering all the wealth quintiles instead of using only the poorest and richest quintiles. Furthermore, the adoption of most recent NFHS dataset, a nationally representative dataset indicates that the estimates are more reliable and comparable. Lastly, the relationship between governance indicators and the coverage indices are crucial to indicate potential governance parameters to be targeted to improve the coverage of RMNCH indicators.

The limitations of this study–there was a lack of scope to capture the quality of maternal and child health care interventions or effective coverage accruing to the unavailability of information related to clean delivery, thermal management, active management, follow up of PNC services in NFHS IV. Recall period of around 5 years poses issues related to recall bias.

## 6. Conclusion and policy recommendation

This study makes a crucial contribution in the realm of public health and the findings become particularly important as we embark on a journey towards the attainment of SDGs. The coverage of RMNCH interventions varied across quintiles and state groups. States having better health infrastructure and robust delivery mechanisms have performed well both in terms of overall coverage and quintile wise coverage. Interventions entailing multiple visits to the facility such as ANC4 and DPT3 performed poor amongst the lowest income horizon mainly due to accessibility and availability impediments. Strong correlation is observed between OI and RMNCH indicators. Specific governance indices such as WC and HE were positively correlated with RMNCH indicators.

Based on the above findings, we prescribe following policy suggestions to improve overall utilisation of RMNCH services and reduce inequalities across geographical boundaries and wealth horizons. The underperformance of ANC4 services demands greater attention. In this aspect, the government agencies/ policy makers might consider following policy prescriptions. First, outreach programs related to ANC4 services at grassroot or village levels can be promoted and monitored by the centralised agency to ensure greater reach. Second, ANC4 services can be incorporated as a prerequisite to attain cash incentives under the existing programmatic interventions (such as JSY). Findings of this study also illuminates that interventions entailing multiple visits (ANC4 and DPT3) should be rendered greater attention. Strong and transparent governance, usage of technologically sound interventions to track mother and child can be promoted. In this journey, the central government might consider supporting state governments grappling with limited resources. Strong and significant correlation between governance indicator and coverage indices reinforces the need to model the dimensions of RMNCH coverage and governance simultaneously to increase the coverage of maternal and child healthcare services and reducing inequalities in its distribution. Finally, the state should increase the proportion of GSDP on health and education.

## 7. Future research

Future research might focus on conducting a comprehensive study measuring the levels of inequality at district level. In addition to wealth indicators, studies might consider probing into the interaction of wealth and place of residence (urban/ rural) to suggest more intricate policies. Future studies can delve deeper into the other dimensions of health inequality like social and ethnic parameters to draw a holistic picture of the disparities existing across different contours of the society. Finally, it would be quite informative to enumerate the level of inequity in the utilisation of child healthcare services.

## Supporting information

S1 Data Detailed description of themes and governance index. (DOCX)

S2 Data Detailed description of RMNCH indicators. (DOCX)

S3 Data Input table for CCI. (DOCX)

S4 Data Theme-wise governance index values across major Indian states. (DOCX)

S5 Data Correlation matrix of CCI, Co-Coverage index and governance indicators. (DOCX)
