# Supplementary material for: Level of inequality and the role of governance indicators in the coverage of reproductive maternal and child healthcare services: Findings from India
Source: PLoS One. 2021 Nov 12;16(11):e0258244. doi: 10.1371/journal.pone.0258244 (PMC8589169; doi:10.1371/journal.pone.0258244)
Supplement: S1 Data — (DOCX) [file pone.0258244.s001.docx]

**S1. Detailed Description of Themes and Governance Index**

1. Essential Infrastructure
2. Power
3. T & D Losses
4. Per Capita Consumption of Power
5. Households electrified as a % of total
6. % of Households with access to safe drinking water
7. Water
8. Total Irrigated Area vs Total Agricultural Area
9. Existence of Water Regulatory Commision
10. Existence of Ground Water Regulation Act
11. Roads and Communication
12. Surface Roads as a % of total Roads
13. Road Density per 1000 sq. KM
14. % of households with access to Cellphone
15. Housing
16. No. of Pucca Houses as a % of total
17. Slum Population as a % of total Urban population
18. % of households with toilets inside premises
19. Support to Human Development
20. Education
21. Educational Development Index
22. ASER Learning Levels
23. No. of Higher Education Colleges per 1 Lakh Population
24. Educational Expenditure as a % of GSDP
25. Health
26. IMR
27. Average Population served per hospital bed
28. Full Immunization
29. Health Exp as a % of GSDP
30. Social Protection
31. Public Distribution System
32. Allocation and offtake of grain under PDS
33. Social Justice and Empowerment
34. % of Pension beneficiaries of the total population above 60
35. % of Households with no land
36. Incidence of crime against SC/ST
37. Minority welfare
38. No. of Minority Children given pre matric scholarship
39. Employment
40. Unemployment Rate
41. % of Manual Casual Labour
42. Women and Children
43. Child
44. Crime against Children
45. Percentage of Child Labour
46. % of Beneficiaries under ICDS
47. Child Sex Ratio
48. % of Malnourished children
49. Women
50. Women Working Population ratio
51. Utilization of Janani Suraksha Yojna Funds
52. Male Female Literacy Gap
53. Institutional Delivery
54. Crime, Law and Order
55. Violent Crimes
56. Rapes per ten lakh population
57. Murders per ten lakh population
58. Dowry Deaths per ten lakh population
59. Atrocities
60. Custodial Deaths per ten lakh population
61. Policing
62. No. of police firings
63. No. of police personnel per ten lakh
64. Delivery of Justice
65. Pendency of Cases
66. Pendency in High Court
67. Pendency in District Court
68. Vacancies of presiding officers
69. Vacancy in High Court
70. Vacancy in District Court
71. Enviroment
72. Pollution and environmental violations
73. Suspended Particulate Matter
74. SO Emissions
75. NO Emissions
76. No. of Environmental Violations in the State (Per capita)
77. Forest cover
78. Increase / Decrease in Forest Cover
79. Renewable energy
80. Renewable Energy as a % of total energy generated
81. Transparency and accountability
82. Transparency
83. Adherence to Section 4 RTI
84. RTPS Act legislated or not
85. No. of Services provided under e-Governance plan
86. Accountability
87. Lok Ayukt: Constituted / Bill Passed, Individual Websites and Chairpersons’ appointment
88. No. of ACB cases disposed as a % of total cases registered
89. Social Audit under NREGA: % of GPs covered
90. Panchayat Devolution Index Score
91. Fiscal Management
92. FRBM indicators
93. Revenue Surplus / Deficit (% of GSDP)
94. Fiscal Surplus / Deficit (% of GSDP)
95. Debt Burden (% of GSDP)
96. Resource generation and development expenditure
97. Per Capita Development Expenditure
98. States own tax revenue growth
99. Economic freedom
100. No. of Industrial Entrepreneurs Memorandum filed
101. Ease of Doing Business
102. Value of MSMEs assets (% of GSDP)

**Steps involved in calculation of Overall Governance Index (OI)**

PAI data were extracted from various union governmental ministries & departments, except the data on underweight children and educational learning levels which was derived from UNICEF and ASER reports. Overall indicator representing governance parameter was derived through a rigorous process. Ranking of the states was done using a three-stage aggregation process. Firstly, a minimum value was subtracted from Indicator’s value and divided by its range, where range is the difference between the maximum and the minimum value. For the variables, which indicated lower values as the better, slightly different methodology was adopted. In such cases, indicator’s value was subtracted from its maximum value and divided by its range, values for each of the binary variables were allotted 0 for no and 1 for yes. Secondly, the values of each of these indicators were summated using weighted aggregation technique which entailed two important considerations- the extent of dominance the state holds on each of the variable and the amount of impact each variable exerts on societal wellbeing. And finally, all the ten themes were given equal weights of 0.1 to arrive at the overall index value.
