# Supplementary material for: Level of inequality and the role of governance indicators in the coverage of reproductive maternal and child healthcare services: Findings from India
Source: PLoS One. 2021 Nov 12;16(11):e0258244. doi: 10.1371/journal.pone.0258244 (PMC8589169; doi:10.1371/journal.pone.0258244)
Supplement: S2 Data — (DOCX) [file pone.0258244.s002.docx]

**S1. Detailed Description of RMNCH Indicators**

| **Indicators used to compute CCI** | **Definition** | **Numerator** | **Denominator** |
| --- | --- | --- | --- |
| Demand for family planning satisfied with modern methods | Percentage of women of reproductive age (15-49 years) who have their need for family planning satisfied with modern methods | Number of women of reproductive age (15-49 years) who have their need for family planning satisfied with modern methods. | Total number of women of reproductive age in need of family planning |
| Antenatal Care | Percentage of women attended 4 or more times during pregnancy by skilled provider | Number of women ages 15-49 years who were attended four or more time during the pregnancy that led to their last birth in last 5 years preceding the survey by skilled provider |  |
| Skilled Birth Attendant | Percentage of live births attended by Skilled Health Personnel | Number of Live births to women ages 15-49 years in the 5 years prior to the survey who were attended during delivery by skilled health personnel | Total number of live births to women ages 15–49 years in the 5 years preceding the survey |
| Immunized with three doses of diphtheria–tetanus–pertussis | Percentage of infants who received three doses of diphtheria–tetanus– pertussis vaccine | Number of surviving infants who received three doses of diphtheria with tetanus toxoid and pertussis containing vaccine | Total number of surviving infants |
|  |  |  |  |
| Immunized against measles (first dose) | Percentage of surviving infants who received the first dose of measles containing vaccine | Number of surviving infants who received the first dose of measles containing vaccine by their first birthday (or as recommended in the national immunization schedule) | Total number of surviving infants |
|  |  |  |  |
|  |  |  |  |
| Immunized with BCG | Percentage of surviving infants who received BCG vaccination | Number of surviving infants who received measles vaccination | Total number of surviving infants |
|  |  |  |  |
| Care seeking for symptoms of pneumonia | Percentage of children ages 0–59 months with suspected pneumonia taken to a health care provider | Number of children ages 0–59 months with symptoms of pneumonia (cough with fast breathing due to problem in the chest or problem in the chest and blocked nose) in the two weeks prior to the survey who were taken to a health care provider) | Total number of children ages 0–59 months with symptoms of pneumonia (cough with fast breathing due to problem in the chest or problem in the chest and blocked nose) in the two weeks prior to the survey |
| ORT treatment of diarrhoea | Percent of children age 0-59 months with diarrhoea in the previous 2 weeks who were given oral rehydration salts (from a packet or pre-package solution) or an appropriate homemade solution (ORT) | Number of children ages 0–59 months with diarrhoea in the two weeks prior to the survey receiving low osmolarity oral rehydration salts and zinc | Total number of children ages 0–59 months with diarrhoea in the two weeks prior to the survey |

**Descriptive for CCI**

| **Interventions** | **Numerator** | **Denominator** | **Proportion** |
| --- | --- | --- | --- |
| BCG vaccination | 56119 | 61277 | 0.92 |
| Pneumonia | 3849 | 4938 | 0.78 |
| Family Panning | 255622 | 349576 | 0.73 |
| Diarrhea | 8346 | 15359 | 0.54 |
| Adequate ANC | 94541 | 183103 | 0.52 |
| DPT | 48250 | 61277 | 0.79 |
| Measles | 49968 | 61277 | 0.82 |
| SBA | 151342 | 188493 | 0.8 |
