# Supplementary material for: Level of inequality and the role of governance indicators in the coverage of reproductive maternal and child healthcare services: Findings from India
Source: PLoS One. 2021 Nov 12;16(11):e0258244. doi: 10.1371/journal.pone.0258244 (PMC8589169; doi:10.1371/journal.pone.0258244)
Supplement: S3 Data — (DOCX) [file pone.0258244.s003.docx]

| **S3:Input Table for CCI** | | | | | | | | | |
| --- | --- | --- | --- | --- | --- | --- | --- | --- | --- |
|  | **At least 4 ANC** | **SBA** | **BCG4** | **FP** | **DPT3** | **Measles** | **% ORS** | **Pneumonia** | **CCI** |
| **India** | | | | | | | | | |
| Poorest | 0.25 | 0.66 | 0.86 | 0.63 | 0.70 | 0.73 | 0.48 | 0.70 | 0.60 |
| Poor | 0.45 | 0.80 | 0.91 | 0.72 | 0.77 | 0.79 | 0.52 | 0.74 | 0.69 |
| Middle | 0.58 | 0.88 | 0.93 | 0.76 | 0.81 | 0.84 | 0.55 | 0.81 | 0.75 |
| Rich | 0.66 | 0.93 | 0.95 | 0.76 | 0.84 | 0.86 | 0.60 | 0.85 | 0.79 |
| Richest | 0.74 | 0.96 | 0.96 | 0.76 | 0.86 | 0.89 | 0.67 | 0.90 | 0.82 |
| Total | 0.01 | 0.83 | 0.92 | 0.73 | 0.79 | 0.82 | 0.54 | 0.78 | 0.66 |
| **Jammu & Kashmir** | | | | | | | | | |
| Poorest | 0.53 | 0.60 | 0.81 | 0.58 | 0.71 | 0.66 | 0.67 | 0.81 | 0.65 |
| Poor | 0.76 | 0.82 | 0.95 | 0.62 | 0.85 | 0.84 | 0.66 | 0.79 | 0.75 |
| Middle | 0.86 | 0.91 | 0.98 | 0.66 | 0.92 | 0.89 | 0.77 | 0.78 | 0.81 |
| Rich | 0.89 | 0.96 | 0.96 | 0.69 | 0.91 | 0.88 | 0.83 | 0.85 | 0.84 |
| Richest | 0.91 | 0.98 | 0.99 | 0.69 | 0.94 | 0.93 | 0.68 | 0.87 | 0.84 |
| Total | 0.83 | 0.89 | 0.95 | 0.66 | 0.89 | 0.86 | 0.72 | 0.81 | 0.80 |
| **Himachal** | | | | | | | | | |
| Poorest | 0.41 | 0.53 | 0.93 | 0.70 | 0.78 | 0.78 | 0.66 | 1.00 | 0.71 |
| Poor | 0.46 | 0.49 | 0.94 | 0.79 | 0.88 | 0.83 | 0.91 | 0.65 | 0.73 |
| Middle | 0.55 | 0.70 | 0.92 | 0.78 | 0.86 | 0.88 | 0.48 | 0.73 | 0.72 |
| Rich | 0.73 | 0.84 | 0.96 | 0.72 | 0.85 | 0.90 | 0.62 | 1.00 | 0.80 |
| Richest | 0.86 | 0.93 | 0.94 | 0.67 | 0.87 | 0.87 | 0.81 | 1.00 | 0.84 |
| Total | 0.70 | 0.80 | 0.94 | 0.72 | 0.86 | 0.88 | 0.70 | 0.92 | 0.79 |
| **Punjab** | | | | | | | | | |
| Poorest | 0.47 | 0.76 | 0.93 | 0.85 | 0.93 | 0.93 | 0.54 | 1.00 | 0.79 |
| Poor | 0.54 | 0.82 | 1.00 | 0.84 | 0.89 | 0.91 | 0.70 | 0.87 | 0.81 |
| Middle | 0.57 | 0.89 | 0.96 | 0.85 | 0.91 | 0.91 | 0.69 | 0.94 | 0.83 |
| Rich | 0.63 | 0.93 | 0.98 | 0.81 | 0.93 | 0.92 | 0.68 | 0.97 | 0.84 |
| Richest | 0.74 | 0.98 | 0.98 | 0.80 | 0.97 | 0.95 | 0.76 | 0.96 | 0.87 |
| Total | 0.68 | 0.95 | 0.98 | 0.81 | 0.95 | 0.94 | 0.73 | 0.96 | 0.86 |
| **Uttaranchal** | | | | | | | | | |
| Poorest | 0.12 | 0.49 | 0.83 | 0.70 | 0.59 | 0.62 | 0.58 | 0.42 | 0.54 |
| Poor | 0.13 | 0.56 | 0.91 | 0.74 | 0.76 | 0.74 | 0.54 | 0.55 | 0.61 |
| Middle | 0.21 | 0.68 | 0.91 | 0.70 | 0.78 | 0.82 | 0.60 | 0.78 | 0.66 |
| Rich | 0.33 | 0.80 | 0.92 | 0.71 | 0.80 | 0.81 | 0.63 | 0.90 | 0.72 |
| Richest | 0.54 | 0.92 | 0.97 | 0.73 | 0.92 | 0.92 | 0.63 | 0.92 | 0.79 |
| Total | 0.31 | 0.74 | 0.92 | 0.72 | 0.81 | 0.82 | 0.60 | 0.77 | 0.69 |
| **Haryana** | | | | | | | | | |
| Poorest | 0.13 | 0.43 | 0.71 | 0.47 | 0.35 | 0.41 | 0.43 | 0.68 | 0.44 |
| Poor | 0.22 | 0.69 | 0.77 | 0.71 | 0.50 | 0.55 | 0.50 | 0.79 | 0.60 |
| Middle | 0.40 | 0.82 | 0.88 | 0.82 | 0.75 | 0.75 | 0.69 | 0.84 | 0.74 |
| Rich | 0.42 | 0.87 | 0.94 | 0.81 | 0.78 | 0.80 | 0.60 | 0.77 | 0.74 |
| Richest | 0.56 | 0.95 | 0.97 | 0.84 | 0.82 | 0.86 | 0.76 | 0.82 | 0.81 |
| Total | 0.45 | 0.87 | 0.92 | 0.82 | 0.76 | 0.79 | 0.67 | 0.80 | 0.75 |
| **Rajasthan** | | | | | | | | | |
| Poorest | 0.22 | 0.76 | 0.79 | 0.69 | 0.58 | 0.64 | 0.59 | 0.81 | 0.63 |
| Poor | 0.30 | 0.85 | 0.86 | 0.74 | 0.67 | 0.76 | 0.55 | 0.90 | 0.69 |
| Middle | 0.37 | 0.90 | 0.91 | 0.76 | 0.77 | 0.83 | 0.62 | 0.96 | 0.75 |
| Rich | 0.48 | 0.94 | 0.94 | 0.75 | 0.81 | 0.85 | 0.61 | 0.84 | 0.76 |
| Richest | 0.61 | 0.97 | 0.96 | 0.78 | 0.83 | 0.90 | 0.75 | 0.95 | 0.83 |
| Total | 0.39 | 0.88 | 0.89 | 0.75 | 0.72 | 0.79 | 0.62 | 0.89 | 0.73 |
| **Uttar Pradesh** | | | | | | | | | |
| Poorest | 0.11 | 0.61 | 0.81 | 0.42 | 0.56 | 0.63 | 0.33 | 0.72 | 0.49 |
| Poor | 0.20 | 0.71 | 0.87 | 0.47 | 0.66 | 0.70 | 0.36 | 0.72 | 0.55 |
| Middle | 0.29 | 0.75 | 0.90 | 0.51 | 0.72 | 0.75 | 0.40 | 0.80 | 0.60 |
| Rich | 0.38 | 0.81 | 0.92 | 0.55 | 0.74 | 0.77 | 0.49 | 0.81 | 0.65 |
| Richest | 0.59 | 0.90 | 0.95 | 0.61 | 0.82 | 0.84 | 0.55 | 0.87 | 0.73 |
| Total | 0.26 | 0.72 | 0.87 | 0.50 | 0.67 | 0.71 | 0.40 | 0.76 | 0.58 |
| **Bihar** | | | | | | | | | |
| Poorest | 0.08 | 0.65 | 0.89 | 0.48 | 0.75 | 0.75 | 0.49 | 0.69 | 0.55 |
| Poor | 0.15 | 0.76 | 0.92 | 0.53 | 0.83 | 0.81 | 0.47 | 0.63 | 0.60 |
| Middle | 0.22 | 0.84 | 0.96 | 0.57 | 0.87 | 0.87 | 0.54 | 0.75 | 0.66 |
| Rich | 0.36 | 0.89 | 0.95 | 0.59 | 0.90 | 0.86 | 0.53 | 0.71 | 0.69 |
| Richest | 0.53 | 0.96 | 0.96 | 0.61 | 0.85 | 0.89 | 0.74 | 0.55 | 0.72 |
| Total | 0.14 | 0.72 | 0.91 | 0.52 | 0.79 | 0.79 | 0.49 | 0.68 | 0.59 |
| **Sikkim** | | | | | | | | | |
| Poorest | 0.58 | 0.79 | 1.00 | 0.96 | 1.00 | 1.00 | 0.00 | 0.00 | 0.66 |
| Poor | 0.61 | 0.89 | 1.00 | 0.64 | 1.00 | 0.80 | 1.00 | 0.00 | 0.71 |
| Middle | 0.80 | 0.98 | 0.99 | 0.72 | 0.93 | 0.93 | 0.92 | 1.00 | 0.88 |
| Rich | 0.79 | 0.99 | 0.99 | 0.65 | 0.92 | 0.94 | 0.74 | 0.00 | 0.71 |
| Richest | 0.80 | 1.00 | 0.94 | 0.60 | 0.85 | 0.94 | 0.00 | 0.00 | 0.60 |
| Total | 0.78 | 0.98 | 0.99 | 0.67 | 0.93 | 0.93 | 0.87 | 1.00 | 0.86 |
| **Arunachal Pradesh** | | | | | | | | | |
| Poorest | 0.12 | 0.23 | 0.54 | 0.04 | 0.35 | 0.36 | 0.51 | 0.35 | 0.26 |
| Poor | 0.22 | 0.42 | 0.68 | 0.06 | 0.53 | 0.55 | 0.70 | 0.47 | 0.39 |
| Middle | 0.33 | 0.67 | 0.76 | 0.06 | 0.58 | 0.65 | 0.68 | 0.46 | 0.44 |
| Rich | 0.41 | 0.88 | 0.83 | 0.04 | 0.62 | 0.63 | 0.83 | 0.73 | 0.54 |
| Richest | 0.40 | 0.92 | 0.86 | 0.03 | 0.67 | 0.72 | 0.42 | 1.00 | 0.53 |
| Total | 0.28 | 0.57 | 0.71 | 0.50 | 0.52 | 0.55 | 0.68 | 0.51 | 0.52 |
| **Nagaland** | | | | | | | | | |
| Poorest | 0.01 | 0.15 | 0.38 | 0.32 | 0.26 | 0.26 | 0.28 | 0.15 | 0.23 |
| Poor | 0.05 | 0.30 | 0.71 | 0.41 | 0.49 | 0.48 | 0.40 | 0.47 | 0.39 |
| Middle | 0.15 | 0.56 | 0.76 | 0.51 | 0.61 | 0.55 | 0.50 | 0.05 | 0.44 |
| Rich | 0.32 | 0.73 | 0.83 | 0.46 | 0.66 | 0.69 | 0.62 | 0.71 | 0.59 |
| Richest | 0.57 | 0.85 | 0.90 | 0.45 | 0.81 | 0.80 | 0.85 | 0.00 | 0.60 |
| Total | 0.15 | 0.44 | 0.69 | 0.44 | 0.53 | 0.51 | 0.47 | 0.33 | 0.43 |
| **Manipur** | | | | | | | | | |
| Poorest | 0.29 | 0.41 | 0.71 | 0.24 | 0.38 | 0.39 | 0.63 | 0.19 | 0.37 |
| Poor | 0.55 | 0.67 | 0.87 | 0.25 | 0.68 | 0.65 | 0.65 | 0.42 | 0.53 |
| Middle | 0.78 | 0.90 | 0.96 | 0.24 | 1.00 | 0.81 | 0.68 | 0.60 | 0.67 |
| Rich | 0.86 | 0.97 | 0.98 | 0.23 | 0.96 | 0.93 | 0.67 | 0.58 | 0.68 |
| Richest | 0.92 | 0.99 | 0.99 | 0.21 | 0.93 | 0.94 | 0.66 | 0.13 | 0.63 |
| Total | 0.69 | 0.81 | 0.91 | 0.24 | 0.78 | 0.75 | 0.66 | 0.44 | 0.59 |
| **Mizoram** | | | | | | | | | |
| Poorest | 0.11 | 0.27 | 0.41 | 0.35 | 0.31 | 0.28 | 0.53 | 1.00 | 0.41 |
| Poor | 0.35 | 0.61 | 0.65 | 0.58 | 0.53 | 0.53 | 0.68 | 0.32 | 0.53 |
| Middle | 0.53 | 0.83 | 0.78 | 0.67 | 0.70 | 0.71 | 0.67 | 0.25 | 0.63 |
| Rich | 0.70 | 0.97 | 0.75 | 0.67 | 0.60 | 0.62 | 0.75 | 0.50 | 0.69 |
| Richest | 0.85 | 0.99 | 0.89 | 0.66 | 0.72 | 0.72 | 0.95 | 0.85 | 0.81 |
| Total | 0.62 | 0.85 | 0.76 | 0.64 | 0.63 | 0.63 | 0.74 | 0.57 | 0.67 |
| **Tripura** | | | | | | | | | |
| Poorest | 0.5 | 0.6 | 0.7 | 0.5 | 0.6 | 0.5 | 0.4 | 0.2 | 0.5 |
| Poor | 0.6 | 0.8 | 0.8 | 0.6 | 0.7 | 0.7 | 0.6 | 0.5 | 0.7 |
| Middle | 0.8 | 0.9 | 0.9 | 0.6 | 0.8 | 0.7 | 0.5 | 1.0 | 0.7 |
| Rich | 0.8 | 1.0 | 1.0 | 0.5 | 0.8 | 0.9 | 1.0 | 1.0 | 0.8 |
| Richest | 0.9 | 1.0 | 1.0 | 0.5 | 0.8 | 0.9 | 1.0 | 0.4 | 0.7 |
| Total | 0.7 | 0.8 | 0.8 | 0.6 | 0.7 | 0.7 | 0.5 | 0.5 | 0.6 |
| **Meghalaya** | | | | | | | | | |
| Poorest | 0.29 | 0.32 | 0.72 | 0.35 | 0.59 | 0.55 | 0.64 | 0.57 | 0.47 |
| Poor | 0.44 | 0.46 | 0.85 | 0.47 | 0.72 | 0.72 | 0.81 | 0.73 | 0.61 |
| Middle | 0.60 | 0.68 | 0.86 | 0.51 | 0.76 | 0.72 | 0.81 | 0.82 | 0.68 |
| Rich | 0.81 | 0.89 | 0.98 | 0.55 | 0.84 | 0.84 | 0.82 | 0.97 | 0.79 |
| Richest | 0.83 | 0.98 | 1.00 | 0.57 | 0.97 | 0.95 | 0.86 | 0.75 | 0.81 |
| Total | 0.52 | 0.57 | 0.85 | 0.49 | 0.73 | 0.71 | 0.79 | 0.76 | 0.64 |
| **Assam** | | | | | | | | | |
| Poorest | 0.33 | 0.57 | 0.77 | 0.57 | 0.56 | 0.62 | 0.48 | 0.48 | 0.53 |
| Poor | 0.45 | 0.78 | 0.81 | 0.58 | 0.67 | 0.72 | 0.62 | 0.64 | 0.63 |
| Middle | 0.57 | 0.89 | 0.90 | 0.54 | 0.80 | 0.82 | 0.57 | 0.71 | 0.68 |
| Rich | 0.67 | 0.95 | 0.90 | 0.53 | 0.81 | 0.85 | 0.75 | 0.48 | 0.70 |
| Richest | 0.77 | 0.99 | 0.96 | 0.51 | 0.87 | 0.88 | 0.81 | 1.00 | 0.80 |
| Total | 0.47 | 0.77 | 0.83 | 0.56 | 0.68 | 0.73 | 0.58 | 0.62 | 0.63 |
| **West Bengal** | | | | | | | | | |
| Poorest | 0.67 | 0.69 | 0.97 | 0.79 | 0.93 | 0.91 | 0.70 | 0.74 | 0.78 |
| Poor | 0.76 | 0.80 | 0.97 | 0.76 | 0.92 | 0.94 | 0.61 | 0.73 | 0.79 |
| Middle | 0.80 | 0.91 | 0.99 | 0.70 | 0.95 | 0.94 | 0.67 | 0.79 | 0.81 |
| Rich | 0.86 | 0.96 | 0.99 | 0.68 | 0.94 | 0.94 | 0.60 | 0.85 | 0.82 |
| Richest | 0.91 | 0.99 | 0.93 | 0.67 | 0.86 | 0.89 | 0.90 | 1.00 | 0.86 |
| Total | 0.77 | 0.84 | 0.97 | 0.73 | 0.93 | 0.93 | 0.66 | 0.77 | 0.80 |
| **Jharkhand** | | | | | | | | | |
| Poorest | 0.17 | 0.59 | 0.93 | 0.58 | 0.78 | 0.80 | 0.44 | 0.53 | 0.57 |
| Poor | 0.32 | 0.80 | 0.96 | 0.69 | 0.86 | 0.84 | 0.48 | 0.79 | 0.69 |
| Middle | 0.40 | 0.83 | 0.99 | 0.70 | 0.88 | 0.88 | 0.55 | 0.68 | 0.71 |
| Rich | 0.56 | 0.93 | 0.99 | 0.69 | 0.91 | 0.92 | 0.63 | 0.83 | 0.77 |
| Richest | 0.70 | 0.97 | 0.99 | 0.66 | 0.90 | 0.90 | 0.65 | 0.78 | 0.78 |
| Total | 0.30 | 0.72 | 0.95 | 0.65 | 0.83 | 0.83 | 0.49 | 0.66 | 0.65 |
| **Odisha** | | | | | | | | | |
| Poorest | 0.55 | 0.79 | 0.92 | 0.64 | 0.86 | 0.85 | 0.71 | 0.59 | 0.71 |
| Poor | 0.64 | 0.90 | 0.94 | 0.67 | 0.89 | 0.88 | 0.70 | 0.78 | 0.77 |
| Middle | 0.68 | 0.95 | 0.96 | 0.65 | 0.93 | 0.92 | 0.77 | 0.72 | 0.79 |
| Rich | 0.70 | 0.95 | 0.94 | 0.62 | 0.88 | 0.90 | 0.82 | 0.77 | 0.78 |
| Richest | 0.75 | 0.96 | 0.96 | 0.62 | 0.93 | 0.93 | 0.93 | 0.75 | 0.81 |
| Total | 0.63 | 0.88 | 0.93 | 0.65 | 0.89 | 0.88 | 0.73 | 0.69 | 0.75 |
| **Chhattisgarh** | | | | | | | | | |
| Poorest | 0.47 | 0.67 | 0.98 | 0.76 | 0.88 | 0.92 | 0.69 | 0.70 | 0.73 |
| Poor | 0.59 | 0.79 | 0.99 | 0.83 | 0.92 | 0.92 | 0.78 | 0.73 | 0.80 |
| Middle | 0.64 | 0.84 | 0.98 | 0.82 | 0.94 | 0.97 | 0.74 | 0.96 | 0.84 |
| Rich | 0.66 | 0.92 | 0.98 | 0.82 | 0.95 | 0.99 | 0.78 | 0.72 | 0.83 |
| Richest | 0.78 | 0.97 | 0.98 | 0.79 | 0.96 | 0.97 | 0.72 | 0.85 | 0.85 |
| Total | 0.59 | 0.80 | 0.98 | 0.80 | 0.92 | 0.94 | 0.74 | 0.76 | 0.80 |
| **Madhya Pradesh** | | | | | | | | | |
| Poorest | 0.20 | 0.64 | 0.86 | 0.79 | 0.64 | 0.73 | 0.55 | 0.68 | 0.64 |
| Poor | 0.31 | 0.80 | 0.93 | 0.80 | 0.73 | 0.80 | 0.60 | 0.73 | 0.70 |
| Middle | 0.41 | 0.87 | 0.94 | 0.80 | 0.78 | 0.82 | 0.59 | 0.79 | 0.74 |
| Rich | 0.49 | 0.90 | 0.97 | 0.77 | 0.82 | 1.00 | 0.61 | 0.84 | 0.77 |
| Richest | 0.65 | 0.95 | 0.97 | 0.76 | 0.89 | 0.90 | 0.66 | 0.84 | 0.81 |
| Total | 0.36 | 0.80 | 0.92 | 0.79 | 0.74 | 0.80 | 0.59 | 7.88 | 0.61 |
| **Gujarat** | | | | | | | | | |
| Poorest | 0.40 | 0.69 | 0.70 | 0.65 | 0.46 | 0.52 | 0.41 | 0.83 | 0.59 |
| Poor | 0.56 | 0.80 | 0.82 | 0.72 | 0.62 | 0.68 | 0.44 | 0.74 | 0.67 |
| Middle | 0.71 | 0.88 | 0.89 | 0.73 | 0.72 | 0.71 | 0.39 | 0.80 | 0.72 |
| Rich | 0.80 | 0.93 | 0.91 | 0.68 | 0.80 | 0.81 | 0.47 | 0.74 | 0.75 |
| Richest | 0.86 | 0.97 | 0.95 | 0.64 | 0.86 | 0.89 | 0.60 | 1.00 | 0.81 |
| Total | 0.71 | 0.88 | 0.88 | 0.68 | 0.73 | 0.75 | 0.46 | 0.81 | 0.72 |
| **Maharashtra** | | | | | | | | | |
| Poorest | 0.56 | 0.75 | 0.80 | 0.88 | 0.62 | 0.75 | 0.59 | 0.86 | 0.74 |
| Poor | 0.68 | 0.89 | 0.89 | 0.86 | 0.76 | 0.79 | 0.60 | 0.67 | 0.77 |
| Middle | 0.72 | 0.92 | 0.90 | 0.85 | 0.72 | 0.82 | 0.59 | 0.85 | 0.79 |
| Rich | 0.76 | 0.96 | 0.91 | 0.84 | 0.79 | 0.88 | 0.74 | 0.98 | 0.85 |
| Richest | 0.79 | 0.97 | 0.91 | 0.83 | 0.81 | 0.87 | 0.69 | 0.87 | 0.84 |
| Total | 0.73 | 0.92 | 0.89 | 0.85 | 0.75 | 0.83 | 0.65 | 0.88 | 0.81 |
| **Andhra Pradesh** | | | | | | | | | |
| Poorest | 0.60 | 0.81 | 0.91 | 0.95 | 0.73 | 0.76 | 0.45 | 0.00 | 0.67 |
| Poor | 0.69 | 0.86 | 0.97 | 0.95 | 0.88 | 0.88 | 0.63 | 1.00 | 0.86 |
| Middle | 0.76 | 0.92 | 0.98 | 0.95 | 0.89 | 0.90 | 0.39 | 0.41 | 0.78 |
| Rich | 0.82 | 0.96 | 0.97 | 0.95 | 0.88 | 0.90 | 0.62 | 0.49 | 0.82 |
| Richest | 0.81 | 0.98 | 0.99 | 0.89 | 0.91 | 0.97 | 0.63 | 1.00 | 0.89 |
| Total | 0.76 | 0.93 | 0.97 | 0.94 | 0.88 | 0.90 | 0.51 | 0.67 | 0.82 |
| **Karnataka** | | | | | | | | | |
| Poorest | 0.68 | 0.91 | 0.91 | 0.90 | 0.83 | 0.82 | 0.56 | 1.00 | 0.83 |
| Poor | 0.71 | 0.93 | 0.96 | 0.89 | 0.82 | 0.81 | 0.60 | 0.84 | 0.82 |
| Middle | 0.73 | 0.95 | 0.94 | 0.85 | 0.80 | 0.84 | 0.63 | 0.94 | 0.83 |
| Rich | 0.69 | 0.94 | 0.93 | 0.83 | 0.79 | 0.86 | 0.41 | 0.88 | 0.78 |
| Richest | 0.70 | 0.94 | 0.87 | 0.76 | 0.75 | 0.82 | 0.52 | 1.00 | 0.78 |
| Total | 0.71 | 0.94 | 0.93 | 0.84 | 0.79 | 0.84 | 0.54 | 0.90 | 0.80 |
| **Goa** | | | | | | | | | |
| Poorest | 0.0 | 1.0 | 1.0 | 0.0 | 1.0 | 1.0 | 0.0 | 0.0 | 0.4 |
| Poor | 0.8 | 0.9 | 1.0 | 0.6 | 1.0 | 1.0 | 1.0 | 0.0 | 0.7 |
| Middle | 0.9 | 1.0 | 1.0 | 0.6 | 1.0 | 0.9 | 0.0 | 1.0 | 0.8 |
| Rich | 0.9 | 1.0 | 1.0 | 0.6 | 0.9 | 1.0 | 0.5 | 1.0 | 0.8 |
| Richest | 0.9 | 1.0 | 1.0 | 0.6 | 0.9 | 1.0 | 0.7 | 1.0 | 0.8 |
| Total | 0.9 | 1.0 | 1.0 | 0.6 | 0.9 | 1.0 | 0.6 | 1.0 | 0.8 |
| **Kerala** | | | | | | | | | |
| Poorest | 0.89 | 1.00 | 1.00 | 0.84 | 0.86 | 0.83 | 0.00 | 0.00 | 0.67 |
| Poor | 0.97 | 0.99 | 1.00 | 0.88 | 0.82 | 0.84 | 0.67 | 0.00 | 0.77 |
| Middle | 0.96 | 0.99 | 0.96 | 0.85 | 0.84 | 0.85 | 0.60 | 1.00 | 0.88 |
| Rich | 0.97 | 1.00 | 0.98 | 0.82 | 0.88 | 0.88 | 0.55 | 0.85 | 0.85 |
| Richest | 0.96 | 1.00 | 0.98 | 0.83 | 0.89 | 0.87 | 0.54 | 1.00 | 0.87 |
| Total | 0.97 | 1.00 | 0.98 | 0.79 | 0.86 | 0.86 | 0.56 | 0.95 | 0.85 |
| **Tamil Nadu** | | | | | | | | | |
| Poorest | 0.79 | 0.95 | 0.94 | 0.87 | 0.51 | 0.78 | 0.60 | 0.86 | 0.79 |
| Poor | 0.81 | 0.99 | 0.94 | 0.86 | 0.89 | 0.89 | 0.69 | 0.99 | 0.87 |
| Middle | 0.81 | 0.99 | 0.94 | 0.85 | 0.92 | 0.93 | 0.65 | 0.87 | 0.86 |
| Rich | 0.83 | 1.00 | 0.96 | 0.83 | 0.88 | 0.90 | 0.67 | 0.93 | 0.86 |
| Richest | 0.83 | 1.00 | 0.97 | 0.83 | 0.92 | 0.91 | 0.74 | 0.92 | 0.88 |
| Total | 0.82 | 0.99 | 0.95 | 0.84 | 0.91 | 0.91 | 0.68 | 0.92 | 0.87 |
| **Telangana** | | | | | | | | | |
| Poorest | 0.61 | 0.72 | 0.96 | 0.88 | 0.86 | 0.93 | 0.70 | 0.00 | 0.70 |
| Poor | 0.68 | 0.85 | 0.97 | 0.92 | 0.84 | 0.91 | 0.61 | 0.86 | 0.83 |
| Middle | 0.73 | 0.92 | 0.95 | 0.92 | 0.88 | 0.87 | 0.67 | 0.92 | 0.86 |
| Rich | 0.78 | 0.95 | 0.98 | 0.87 | 0.84 | 0.87 | 0.60 | 0.77 | 0.83 |
| Richest | 0.84 | 0.96 | 1.00 | 0.87 | 0.97 | 0.97 | 0.90 | 0.91 | 0.91 |
| Total | 0.75 | 0.92 | 0.98 | 0.89 | 0.88 | 0.90 | 0.66 | 0.84 | 0.85 |
