# Supplementary material for: Level of inequality and the role of governance indicators in the coverage of reproductive maternal and child healthcare services: Findings from India
Source: PLoS One. 2021 Nov 12;16(11):e0258244. doi: 10.1371/journal.pone.0258244 (PMC8589169; doi:10.1371/journal.pone.0258244)
Supplement: S4 Data — (DOCX) [file pone.0258244.s004.docx]

**S4. Theme-wise Governance Index Values across Major Indian States**

| **States** | **EI** | **HE** | **SP** | **WC** | **CLO** | **DJ** | **TJ** | **ENV** | **FM** | **EF** | **OI** |
| --- | --- | --- | --- | --- | --- | --- | --- | --- | --- | --- | --- |
| **Andhra Pradesh** | 0.660 | 0.454 | 0.370 | 0.503 | 0.360 | 0.427 | 0.449 | 0.551 | 0.388 | 0.505 | 0.467 |
| **Bihar** | 0.414 | 0.220 | 0.303 | 0.404 | 0.537 | 0.327 | 0.513 | 0.391 | 0.473 | 0.121 | 0.370 |
| **Gujarat** | 0.675 | 0.431 | 0.432 | 0.472 | 0.670 | 0.391 | 0.469 | 0.497 | 0.421 | 0.890 | 0.535 |
| **Haryana** | 0.716 | 0.548 | 0.350 | 0.527 | 0.406 | 0.464 | 0.627 | 0.416 | 0.410 | 0.345 | 0.481 |
| **Himachal Pradesh** | 0.714 | 0.724 | 0.600 | 0.610 | 0.653 | 0.459 | 0.674 | 0.497 | 0.248 | 0.210 | 0.539 |
| **Karnataka** | 0.558 | 0.580 | 0.665 | 0.559 | 0.597 | 0.386 | 0.714 | 0.625 | 0.427 | 0.358 | 0.547 |
| **Kerala** | 0.553 | 0.692 | 0.636 | 0.655 | 0.658 | 0.565 | 0.757 | 0.482 | 0.425 | 0.252 | 0.568 |
| **Madhya Pradesh** | 0.387 | 0.266 | 0.603 | 0.483 | 0.499 | 0.445 | 0.571 | 0.433 | 0.450 | 0.384 | 0.452 |
| **Maharashtra** | 0.612 | 0.510 | 0.521 | 0.554 | 0.622 | 0.442 | 0.609 | 0.484 | 0.393 | 0.610 | 0.536 |
| **Odisha** | 0.239 | 0.439 | 0.516 | 0.561 | 0.510 | 0.433 | 0.442 | 0.481 | 0.469 | 0.331 | 0.442 |
| **Punjab** | 0.774 | 0.637 | 0.400 | 0.540 | 0.650 | 0.376 | 0.663 | 0.472 | 0.454 | 0.366 | 0.533 |
| **Rajasthan** | 0.557 | 0.439 | 0.592 | 0.437 | 0.536 | 0.388 | 0.479 | 0.436 | 0.433 | 0.436 | 0.473 |
| **Tamil Nadu** | 0.636 | 0.551 | 0.589 | 0.608 | 0.724 | 0.529 | 0.312 | 0.682 | 0.426 | 0.443 | 0.549 |
| **Uttar Pradesh** | 0.582 | 0.284 | 0.575 | 0.466 | 0.441 | 0.393 | 0.604 | 0.397 | 0.494 | 0.375 | 0.461 |
| **West Bengal** | 0.472 | 0.420 | 0.650 | 0.548 | 0.641 | 0.445 | 0.584 | 0.529 | 0.425 | 0.337 | 0.505 |
| **Telangana** | 0.501 | 0.333 | 0.215 | 0.482 | 0.268 | 0.464 | 0.537 | 0.457 | 0.654 | 0.639 | 0.455 |

Note: EI- Essential Infrastructure, ENV- Environment, SP-Social Protection, HE- Health and Education, DJ- Delivery of Justice, CLO-Crime, Law and Order, WC-Women and Child, TJ-Transparency and Public Accountability, FM- Fiscal Management, EF-Economic Freedom, OI-Overall Index
