# Supplementary material for: Level of inequality and the role of governance indicators in the coverage of reproductive maternal and child healthcare services: Findings from India
Source: PLoS One. 2021 Nov 12;16(11):e0258244. doi: 10.1371/journal.pone.0258244 (PMC8589169; doi:10.1371/journal.pone.0258244)
Supplement: S5 Data — (DOCX) [file pone.0258244.s005.docx]

**S5: Correlation Matrix of CCI, Co-Coverage Index and Governance Indicators**


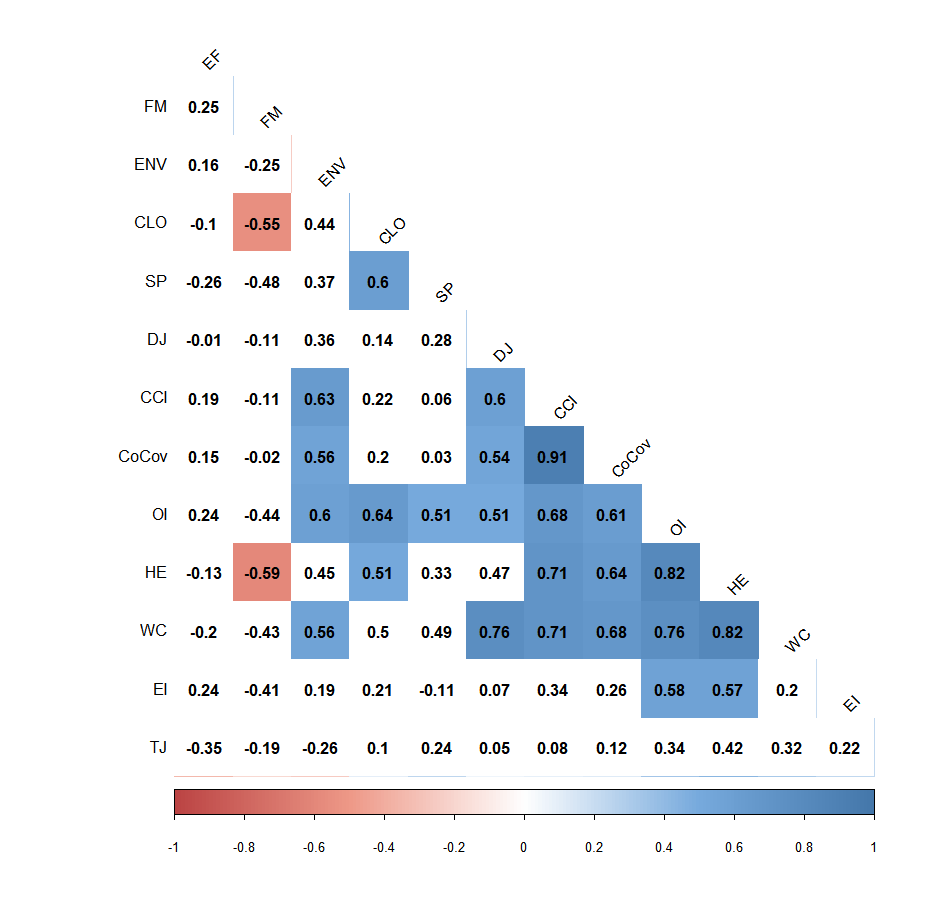


EI- Essential Infrastructure, ENV- Environment, SP-Social Protection, HE- Health and Education, DJ- Delivery of Justice, CLO-Crime, Law and Order, WC-Women and Child, TJ-Transparency and Public Accountability, FM- Fiscal Management , EF-Economic Freedom, OI-Overall Index, CCI-Composite Coverage Index, Co-Cov -Co-Coverage Index
